# 3β-Acet­oxy-6-hy­droxy­imino­cholestane

**DOI:** 10.1107/S1600536811007306

**Published:** 2011-03-05

**Authors:** Kamal Aziz Ketuly, A. Hamid A. Hadi, Seik Weng Ng, Edward R. T. Tiekink

**Affiliations:** a Department of Chemistry, University of Malaya, 50603 Kuala Lumpur, Malaysia

## Abstract

Two independent mol­ecules comprise the asymmetric unit of the title cholestane derivative, C_29_H_49_NO_3_ {systematic name: (3*S*,8*S*,9*S*,10*R*,13*R*,14*S*,17*R*)-17-[(1*R*)-1,5-dimethyl­hex­yl]-6-hy­droxy­imino-10,13-dimethyl-2,3,4,7,8,9,10,11,12,13,14,15,16,17-tetra­deca­hydro-1*H*-cyclo­penta­[*a*]phenanthren-3-yl ace­tate}. The major differences between the mol­ecules relate to the relative orientations of the terminal acetyl [C—C—O—C torsion angles = −158.8 (3) and −81.7 (3)°] and alkyl groups [C—C—C—C = 168.9 (3) and 65.8 (4)°]. In the crystal, the independent mol­ecules associate *via* pairs of O—H⋯N hydrogen bonds, forming dimeric aggregates. Supra­molecular layers in the *ab* plane are mediated by C—H⋯O inter­actions.

## Related literature

For background to this study and further details of the synthetic procedures, see: Ketuly & Hadi (2010 ▶). For previous syntheses, see: Anagnostopoulos & Fieser (1954 ▶); Petersen (1963 ▶); Choucair *et al.* (2004 ▶). For related structures, see: Ketuly *et al.* (1997 ▶, 2010 ▶). For ring conformational analysis, see: Cremer & Pople (1975 ▶).
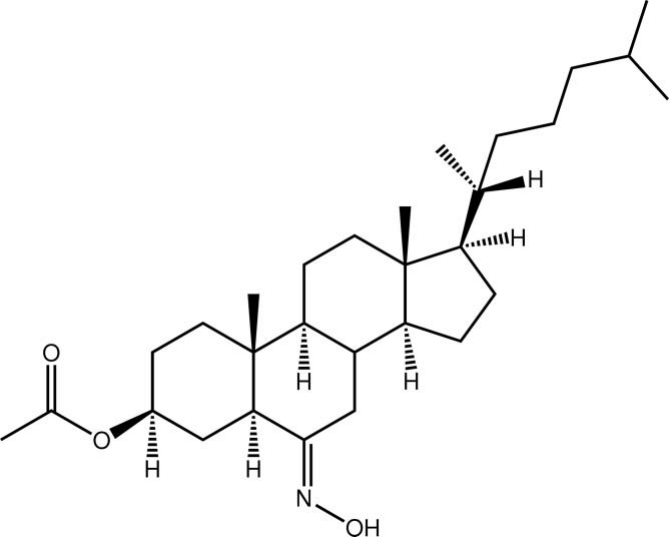


## Experimental

### 

#### Crystal data

C_29_H_49_NO_3_
*M* *_r_* = 459.69
Monoclinic, 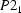

*a* = 11.3934 (13) Å
*b* = 9.6588 (11) Å
*c* = 25.018 (3) Å
β = 93.466 (2)°
*V* = 2748.1 (5) Å^3^
*Z* = 4
Mo *K*α radiation
μ = 0.07 mm^−1^
*T* = 100 K
0.35 × 0.30 × 0.03 mm

#### Data collection

Bruker SMART APEX CCD diffractometer
Absorption correction: multi-scan (*SADABS*; Sheldrick, 1996 ▶) *T*_min_ = 0.757, *T*_max_ = 0.862
26531 measured reflections
6690 independent reflections
4553 reflections with *I* > 2σ(*I*)
*R*_int_ = 0.086

#### Refinement

*R*[*F* ^2^ > 2σ(*F* ^2^)] = 0.050
*wR*(*F* ^2^) = 0.114
*S* = 0.99
6690 reflections
615 parameters
1 restraint
H atoms treated by a mixture of independent and constrained refinement
Δρ_max_ = 0.24 e Å^−3^
Δρ_min_ = −0.23 e Å^−3^
Absolute structure: nd
Flack parameter: ?
Rogers parameter: ?

### 

Data collection: *APEX2* (Bruker, 2009 ▶); cell refinement: *SAINT* (Bruker, 2009 ▶); data reduction: *SAINT*; program(s) used to solve structure: *SHELXS97* (Sheldrick, 2008 ▶); program(s) used to refine structure: *SHELXL97* (Sheldrick, 2008 ▶); molecular graphics: *ORTEP-3* (Farrugia, 1997 ▶), *DIAMOND* (Brandenburg, 2006 ▶) and *Qmol* (Gans & Shalloway, 2001 ▶); software used to prepare material for publication: *publCIF* (Westrip, 2010 ▶).

## Supplementary Material

Crystal structure: contains datablocks general, I. DOI: 10.1107/S1600536811007306/hb5806sup1.cif

Structure factors: contains datablocks I. DOI: 10.1107/S1600536811007306/hb5806Isup2.hkl

Additional supplementary materials:  crystallographic information; 3D view; checkCIF report

## Figures and Tables

**Table 1:** Hydrogen-bond geometry (Å, °)

*D*—H⋯*A*	*D*—H	H⋯*A*	*D*⋯*A*	*D*—H⋯*A*
O1—H1⋯N2	0.98 (4)	1.88 (4)	2.809 (3)	157 (4)
O4—H4⋯N1	0.95 (4)	1.82 (4)	2.733 (3)	160 (3)
C9—H9c⋯O6^i^	0.98	2.58	3.404 (4)	142
C37—H37c⋯O3^ii^	0.98	2.40	3.373 (4)	169

Symmetry codes: (i) 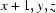; (ii) 
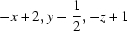
.

**Fig. 1.**
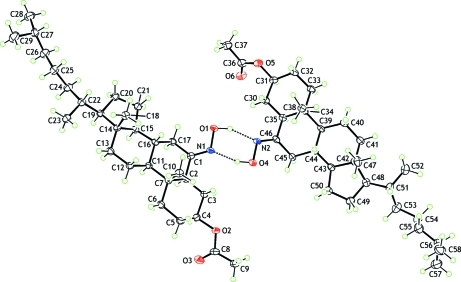
The molecular structures of the two independent molecules comprising the asymmetric unit of (I) showing displacement ellipsoids at the 50% probability level. The molecules are connected into dimeric aggregates via pairs of O—H···N hydrogen bonds (dashed lines).

**Fig. 2.**
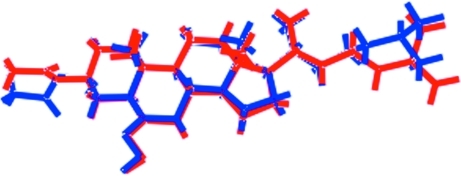
Overlay diagram the two independent molecules comprising the asymmetric unit of (I). The independent molecule having the N1 atom is shown in red.

**Fig. 3.**
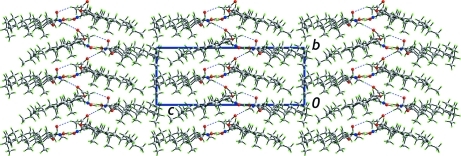
View in projection down the a axis of the crystal packing of (I). The O—H···N hydrogen bonds and C—H···O contacts are shown as orange and blue dashed lines, respectively.

## supplementary crystallographic information

### Comment

The title compound, 3β-Acetoxy-6 N-hydroxyiminocholestane, (I), is a known species and has been utilized as an intermediate for the preparation of 6-ketocholestanol acetate, which it readily affords upon reduction with zinc and acetic acid followed by acid hydrolysis (Anagnostopoulos & Fieser, 1954; Petersen, 1963). Interest in hydroxyimino-steroids stems from a broad investigation into the correlation of structure with biological activity of modified steroid hormones (Choucair *et al.*, 2004). In continuation of systematic structural analyses of related steroidal compounds (Ketuly *et al.*, 1997; Ketuly *et al.*, 2010), the X-ray crystallographic analysis of (I) was conducted.

Two independent molecules comprise the asymmetric unit of (I), Fig. 1. These are linked into dimeric aggregates *via* O—H···N hydrogen bonds, Table 1. From the overlay diagram, Fig. 3, it is evident that the molecules differ in the relative orientations of the terminal acetyl and alkyl substituents. For the former, the different conformation is manifested in the values of the C3—C4—O2—C8 and C30—C31—O5—C36 torsion angles of -158.8 (3) and -81.7 (3) °, respectively. For the alkyl chains, the differences are seen in the C22—C24—C25—C26 and C51—C53—C54—C55 torsion angles of 168.9 (3) and 65.8 (4) °, respectively. Each of the six-membered rings adopts a chair conformation or close to a chair conformation, and each of the five-membered rings has a twisted conformation, about the C14—C15 and C42—C43 bonds, respectively (Cremer & Pople, 1975).

The most notable feature of the crystal packing other than the aforementioned O—H···N hydrogen bonds is the presence of C—H···O interactions, Table 1. These lead to the formation of supramolecular layers in the *ab* plane, Fig. 3.

### Experimental

Hydroxylamine hydrochloride (13.5 mg) was dissolved in dried and purified pyridine (2 ml) and 3β-acetoxy-5α-chloestan-6-one (10 mg) added. The solution mixture was heated at 353 K for 4 h. The solvent was dried under vacuum, yielding crude crystals. Recrystallization from methanol and water (10:1, *v*/*v*) yielded compound (I): yield 9.1 mg, 88%, *M*.pt. 474–475 K. Lit. *M*.pt. 475–476 K (Petersen, 1963). Compound (I) was also isolated as an intermediate byproduct during the reduction of 3β-acetoxy-6-nitrocholest-5-ene to 3β-acetoxy-6-oxo-cholestanol. Thus, 3β-acetoxy-6-nitrocholest-5-ene (5 g, 10.6 mmol) was dissolved in glacial acetic acid (100 ml) and stirred with a Hershbury stirrer and diluted with water (5 ml). Zinc dust (10 g) was added in small portions over a period of 0.5 h. The suspension was then heated under reflux for 4.5 h. The solution was filtered and washed with acetic acid (2 *x* 6.5 ml). The filtrate was diluted with water (100 ml), cooled in an ice-bath and the organic layer was extracted with ether. The yellow viscous product was crystallized from methanol, dried (4.41 g) and recrystallized four times from methanol with a few drops of ether, yielding 3β-acetoxy-6-oxo-cholestanol (3.12 g), *M*.pt. 402–404 K. Lit. 409 K (Choucair *et al.*, 2004). The combined mother liquors were dried and four times recrystallized from methanol and water (10:1, *v*/*v*), yielding colourless plates of (I), (0.24 g), *M*.pt. 474–475 K. The purification and vacuum sublimation methods employed in the study follow literature precedents (Ketuly & Hadi, 2010).

### Refinement

Carbon-bound H-atoms were placed in calculated positions (C—H 0.98 to 1.00 Å) and were included in the refinement in the riding model approximation, with *U*_iso_(H) set to 1.2 to 1.5*U*_equiv_(C). The oxygen-bound H atoms were located from a difference map and refined freely. In the absence of significant anomalous scattering effects, 5428 Friedel pairs were averaged in the final refinement. However, the absolute configuration was assigned on the basis of the known chirality of the 3β-acetoxy-5α-chloestan-6-one starting material. Two reflections, *i.e.* (0 0 1) and (0 0 2), were omitted from the final refinement owing to poor agreement.

### Crystal data

**Table:**

C_29_H_49_NO_3_	*F*(000) = 1016
*M**_r_* = 459.69	*D*_x_ = 1.111 Mg m^−^^3^
Monoclinic, *P*2_1_	Mo *K*α radiation, λ = 0.71073 Å
Hall symbol: P 2yb	Cell parameters from 2461 reflections
*a* = 11.3934 (13) Å	θ = 2.3–20.8°
*b* = 9.6588 (11) Å	µ = 0.07 mm^−^^1^
*c* = 25.018 (3) Å	*T* = 100 K
β = 93.466 (2)°	Plate, colourless
*V* = 2748.1 (5) Å^3^	0.35 × 0.30 × 0.03 mm
*Z* = 4	

### Data collection

**Table:**

Bruker SMART APEX CCD diffractometer	6690 independent reflections
Radiation source: fine-focus sealed tube	4553 reflections with *I* > 2σ(*I*)
graphite	*R*_int_ = 0.086
ω scans	θ_max_ = 27.5°, θ_min_ = 2.0°
Absorption correction: multi-scan (*SADABS*; Sheldrick, 1996)	*h* = −14→14
*T*_min_ = 0.757, *T*_max_ = 0.862	*k* = −11→12
26531 measured reflections	*l* = −32→32

### Refinement

**Table:**

Refinement on *F*^2^	Secondary atom site location: difference Fourier map
Least-squares matrix: full	Hydrogen site location: inferred from neighbouring sites
*R*[*F*^2^ > 2σ(*F*^2^)] = 0.050	H atoms treated by a mixture of independent and constrained refinement
*wR*(*F*^2^) = 0.114	*w* = 1/[σ^2^(*F*_o_^2^) + (0.0489*P*)^2^] where *P* = (*F*_o_^2^ + 2*F*_c_^2^)/3
*S* = 0.99	(Δ/σ)_max_ < 0.001
6690 reflections	Δρ_max_ = 0.24 e Å^−^^3^
615 parameters	Δρ_min_ = −0.23 e Å^−^^3^
1 restraint	Absolute structure: nd
Primary atom site location: structure-invariant direct methods	

### Special details

**Table:**

Geometry. All e.s.d.'s (except the e.s.d. in the dihedral angle between two l.s. planes) are estimated using the full covariance matrix. The cell e.s.d.'s are taken into account individually in the estimation of e.s.d.'s in distances, angles and torsion angles; correlations between e.s.d.'s in cell parameters are only used when they are defined by crystal symmetry. An approximate (isotropic) treatment of cell e.s.d.'s is used for estimating e.s.d.'s involving l.s. planes.
Refinement. Refinement of *F*^2^ against ALL reflections. The weighted *R*-factor *wR* and goodness of fit *S* are based on *F*^2^, conventional *R*-factors *R* are based on *F*, with *F* set to zero for negative *F*^2^. The threshold expression of *F*^2^ > σ(*F*^2^) is used only for calculating *R*-factors(gt) *etc*. and is not relevant to the choice of reflections for refinement. *R*-factors based on *F*^2^ are statistically about twice as large as those based on *F*, and *R*- factors based on ALL data will be even larger.

### Fractional atomic coordinates and isotropic or equivalent isotropic displacement parameters (Å^2^)

**Table:**

	*x*	*y*	*z*	*U*_iso_*/*U*_eq_	
O1	0.84194 (18)	0.5005 (3)	0.55612 (8)	0.0249 (5)	
O2	1.37405 (18)	0.6484 (2)	0.51114 (8)	0.0238 (5)	
O3	1.4744 (2)	0.8086 (3)	0.46763 (10)	0.0369 (7)	
O4	1.08653 (19)	0.4934 (3)	0.62945 (9)	0.0321 (6)	
O5	0.55480 (19)	0.3995 (2)	0.69942 (9)	0.0261 (5)	
O6	0.5309 (2)	0.6211 (3)	0.67381 (10)	0.0321 (6)	
N1	0.9531 (2)	0.5325 (3)	0.53618 (10)	0.0191 (6)	
N2	0.9777 (2)	0.4768 (3)	0.65324 (10)	0.0240 (6)	
C1	0.9457 (3)	0.5799 (3)	0.48875 (12)	0.0182 (7)	
C2	1.0580 (3)	0.6150 (3)	0.46315 (12)	0.0186 (7)	
H2	1.0485	0.7120	0.4495	0.022*	
C3	1.1680 (3)	0.6152 (4)	0.50172 (12)	0.0208 (7)	
H3A	1.1554	0.6770	0.5324	0.025*	
H3B	1.1829	0.5206	0.5157	0.025*	
C4	1.2728 (3)	0.6646 (4)	0.47275 (12)	0.0221 (7)	
H4A	1.2627	0.7643	0.4627	0.027*	
C5	1.2916 (3)	0.5790 (4)	0.42338 (12)	0.0230 (8)	
H5A	1.3142	0.4837	0.4343	0.028*	
H5B	1.3569	0.6192	0.4041	0.028*	
C6	1.1800 (3)	0.5736 (4)	0.38548 (12)	0.0230 (7)	
H6A	1.1947	0.5122	0.3549	0.028*	
H6B	1.1637	0.6676	0.3711	0.028*	
C7	1.0709 (3)	0.5212 (3)	0.41278 (11)	0.0177 (7)	
C8	1.4698 (3)	0.7243 (4)	0.50283 (14)	0.0261 (8)	
C9	1.5689 (3)	0.6876 (4)	0.54238 (14)	0.0322 (9)	
H9A	1.6407	0.7351	0.5328	0.048*	
H9B	1.5817	0.5872	0.5419	0.048*	
H9C	1.5492	0.7163	0.5783	0.048*	
C10	1.0868 (3)	0.3688 (3)	0.42964 (13)	0.0235 (7)	
H10A	1.1521	0.3613	0.4568	0.035*	
H10B	1.1040	0.3130	0.3984	0.035*	
H10C	1.0144	0.3351	0.4445	0.035*	
C11	0.9585 (3)	0.5404 (3)	0.37497 (11)	0.0181 (7)	
H11	0.9568	0.6399	0.3640	0.022*	
C12	0.9603 (3)	0.4552 (3)	0.32305 (12)	0.0209 (7)	
H12A	0.9672	0.3558	0.3324	0.025*	
H12B	1.0307	0.4812	0.3040	0.025*	
C13	0.8498 (3)	0.4763 (4)	0.28501 (12)	0.0226 (7)	
H13A	0.8484	0.5726	0.2715	0.027*	
H13B	0.8537	0.4135	0.2539	0.027*	
C14	0.7370 (3)	0.4477 (3)	0.31316 (12)	0.0190 (7)	
C15	0.7391 (3)	0.5408 (3)	0.36309 (11)	0.0192 (7)	
H15	0.7478	0.6381	0.3502	0.023*	
C16	0.8436 (3)	0.5132 (3)	0.40277 (11)	0.0174 (7)	
H16	0.8419	0.4134	0.4132	0.021*	
C17	0.8359 (3)	0.6006 (4)	0.45354 (12)	0.0210 (7)	
H17A	0.8273	0.6996	0.4439	0.025*	
H17B	0.7665	0.5724	0.4728	0.025*	
C18	0.7281 (3)	0.2929 (3)	0.32761 (13)	0.0239 (8)	
H18A	0.6553	0.2765	0.3455	0.036*	
H18B	0.7956	0.2667	0.3516	0.036*	
H18C	0.7278	0.2372	0.2949	0.036*	
C19	0.6195 (3)	0.4962 (3)	0.28363 (11)	0.0195 (7)	
H19	0.6335	0.5897	0.2680	0.023*	
C20	0.5362 (3)	0.5155 (4)	0.33006 (12)	0.0251 (8)	
H20A	0.4877	0.5999	0.3241	0.030*	
H20B	0.4830	0.4349	0.3321	0.030*	
C21	0.6149 (3)	0.5290 (4)	0.38261 (12)	0.0241 (8)	
H21A	0.5940	0.6125	0.4029	0.029*	
H21B	0.6075	0.4464	0.4056	0.029*	
C22	0.5642 (3)	0.4040 (3)	0.23836 (12)	0.0220 (7)	
H22	0.5499	0.3106	0.2539	0.026*	
C23	0.6450 (3)	0.3848 (4)	0.19192 (12)	0.0268 (8)	
H23A	0.6010	0.3405	0.1617	0.040*	
H23B	0.7120	0.3264	0.2037	0.040*	
H23C	0.6737	0.4754	0.1807	0.040*	
C24	0.4450 (3)	0.4623 (3)	0.21712 (13)	0.0255 (8)	
H24A	0.4027	0.4976	0.2477	0.031*	
H24B	0.4590	0.5416	0.1933	0.031*	
C25	0.3664 (3)	0.3576 (4)	0.18636 (13)	0.0280 (8)	
H25A	0.3645	0.2707	0.2073	0.034*	
H25B	0.4009	0.3363	0.1519	0.034*	
C26	0.2406 (3)	0.4099 (4)	0.17519 (14)	0.0293 (8)	
H26A	0.2134	0.4512	0.2085	0.035*	
H26B	0.2415	0.4843	0.1480	0.035*	
C27	0.1525 (3)	0.3005 (4)	0.15556 (13)	0.0263 (8)	
H27	0.1581	0.2220	0.1816	0.032*	
C28	0.0275 (3)	0.3572 (4)	0.15502 (15)	0.0394 (10)	
H28A	0.0106	0.3874	0.1912	0.059*	
H28B	−0.0283	0.2846	0.1431	0.059*	
H28C	0.0197	0.4361	0.1304	0.059*	
C29	0.1768 (3)	0.2427 (5)	0.10086 (15)	0.0417 (10)	
H29A	0.2546	0.1992	0.1025	0.063*	
H29B	0.1745	0.3180	0.0746	0.063*	
H29C	0.1169	0.1736	0.0902	0.063*	
C30	0.7648 (3)	0.4453 (4)	0.69849 (12)	0.0215 (7)	
H30A	0.7533	0.5199	0.6716	0.026*	
H30B	0.7732	0.3567	0.6792	0.026*	
C31	0.6589 (3)	0.4380 (3)	0.73216 (12)	0.0228 (7)	
H31	0.6462	0.5305	0.7488	0.027*	
C32	0.6743 (3)	0.3301 (3)	0.77571 (12)	0.0242 (8)	
H32A	0.6789	0.2371	0.7593	0.029*	
H32B	0.6053	0.3316	0.7979	0.029*	
C33	0.7860 (3)	0.3571 (4)	0.81116 (12)	0.0242 (8)	
H33A	0.7955	0.2823	0.8381	0.029*	
H33B	0.7770	0.4454	0.8306	0.029*	
C34	0.8974 (3)	0.3646 (3)	0.77964 (12)	0.0196 (7)	
C35	0.8765 (3)	0.4736 (3)	0.73399 (12)	0.0193 (7)	
H35	0.8640	0.5644	0.7520	0.023*	
C36	0.4993 (3)	0.5018 (4)	0.67105 (13)	0.0271 (8)	
C37	0.3957 (3)	0.4475 (4)	0.63748 (14)	0.0316 (9)	
H37A	0.3414	0.5237	0.6282	0.047*	
H37B	0.3551	0.3770	0.6577	0.047*	
H37C	0.4229	0.4063	0.6046	0.047*	
C38	0.9238 (3)	0.2221 (3)	0.75585 (13)	0.0224 (7)	
H38A	0.9534	0.1598	0.7845	0.034*	
H38B	0.9832	0.2320	0.7294	0.034*	
H38C	0.8516	0.1835	0.7385	0.034*	
C39	1.0043 (3)	0.4165 (3)	0.81616 (11)	0.0197 (7)	
H39	0.9850	0.5135	0.8265	0.024*	
C40	1.0249 (3)	0.3368 (4)	0.86832 (12)	0.0277 (8)	
H40A	1.0383	0.2381	0.8599	0.033*	
H40B	0.9529	0.3422	0.8885	0.033*	
C41	1.1297 (3)	0.3902 (4)	0.90420 (12)	0.0265 (8)	
H41A	1.1127	0.4852	0.9164	0.032*	
H41B	1.1406	0.3303	0.9362	0.032*	
C42	1.2428 (3)	0.3912 (3)	0.87421 (12)	0.0220 (7)	
C43	1.2180 (3)	0.4791 (3)	0.82297 (11)	0.0201 (7)	
H43	1.1939	0.5728	0.8353	0.024*	
C44	1.1168 (3)	0.4260 (3)	0.78592 (12)	0.0206 (7)	
H44	1.1372	0.3313	0.7732	0.025*	
C45	1.0981 (3)	0.5218 (4)	0.73689 (11)	0.0229 (7)	
H45A	1.0953	0.6190	0.7492	0.028*	
H45B	1.1658	0.5124	0.7142	0.028*	
C46	0.9867 (3)	0.4894 (3)	0.70391 (11)	0.0201 (7)	
C47	1.2818 (3)	0.2435 (3)	0.86097 (13)	0.0286 (8)	
H47A	1.2210	0.1991	0.8374	0.043*	
H47B	1.2934	0.1899	0.8942	0.043*	
H47C	1.3557	0.2471	0.8429	0.043*	
C48	1.3496 (3)	0.4747 (3)	0.90062 (12)	0.0239 (7)	
H48	1.3176	0.5633	0.9145	0.029*	
C49	1.4219 (3)	0.5117 (4)	0.85183 (12)	0.0265 (8)	
H49A	1.4518	0.6077	0.8552	0.032*	
H49B	1.4898	0.4483	0.8501	0.032*	
C50	1.3397 (3)	0.4970 (4)	0.80088 (12)	0.0254 (8)	
H50A	1.3425	0.5808	0.7782	0.030*	
H50B	1.3611	0.4153	0.7797	0.030*	
C51	1.4265 (3)	0.4091 (4)	0.94657 (12)	0.0270 (8)	
H51	1.4669	0.3263	0.9323	0.032*	
C52	1.3565 (3)	0.3630 (4)	0.99354 (13)	0.0351 (9)	
H52A	1.3037	0.2871	0.9821	0.053*	
H52B	1.3101	0.4410	1.0058	0.053*	
H52C	1.4107	0.3313	1.0229	0.053*	
C53	1.5206 (3)	0.5147 (4)	0.96584 (14)	0.0333 (9)	
H53A	1.5581	0.5514	0.9341	0.040*	
H53B	1.4808	0.5932	0.9826	0.040*	
C54	1.6177 (3)	0.4619 (4)	1.00560 (15)	0.0402 (10)	
H54A	1.5811	0.4213	1.0369	0.048*	
H54B	1.6667	0.5411	1.0185	0.048*	
C55	1.6970 (3)	0.3526 (4)	0.98144 (14)	0.0347 (9)	
H55A	1.6528	0.2646	0.9775	0.042*	
H55B	1.7161	0.3833	0.9452	0.042*	
C56	1.8112 (3)	0.3258 (4)	1.01455 (15)	0.0410 (10)	
H56	1.7915	0.3076	1.0523	0.049*	
C57	1.8932 (3)	0.4494 (5)	1.01459 (17)	0.0476 (11)	
H57A	1.9674	0.4262	1.0345	0.071*	
H57B	1.8565	0.5283	1.0317	0.071*	
H57C	1.9087	0.4735	0.9776	0.071*	
C58	1.8720 (3)	0.1987 (5)	0.99400 (17)	0.0490 (12)	
H58A	1.9453	0.1825	1.0157	0.073*	
H58B	1.8898	0.2132	0.9566	0.073*	
H58C	1.8203	0.1181	0.9965	0.073*	
H1	0.871 (4)	0.480 (5)	0.5931 (17)	0.075 (15)*	
H4	1.057 (3)	0.508 (4)	0.5936 (15)	0.047 (11)*	

### Atomic displacement parameters (Å^2^)

**Table:**

	*U*^11^	*U*^22^	*U*^33^	*U*^12^	*U*^13^	*U*^23^
O1	0.0188 (12)	0.0347 (14)	0.0221 (11)	−0.0064 (11)	0.0073 (10)	0.0009 (11)
O2	0.0167 (12)	0.0278 (14)	0.0271 (12)	−0.0059 (10)	0.0026 (10)	0.0033 (10)
O3	0.0334 (16)	0.0372 (16)	0.0408 (15)	−0.0089 (12)	0.0073 (12)	0.0070 (13)
O4	0.0194 (13)	0.0555 (18)	0.0222 (12)	−0.0054 (13)	0.0060 (10)	0.0065 (12)
O5	0.0205 (12)	0.0240 (13)	0.0341 (12)	0.0005 (10)	0.0049 (10)	−0.0047 (11)
O6	0.0284 (14)	0.0222 (14)	0.0455 (15)	0.0027 (11)	0.0015 (12)	−0.0042 (12)
N1	0.0170 (14)	0.0208 (15)	0.0202 (13)	−0.0027 (12)	0.0059 (11)	−0.0026 (11)
N2	0.0165 (14)	0.0330 (18)	0.0235 (13)	−0.0007 (13)	0.0086 (11)	0.0025 (13)
C1	0.0174 (17)	0.0172 (17)	0.0201 (15)	−0.0003 (13)	0.0026 (13)	−0.0069 (13)
C2	0.0180 (17)	0.0161 (17)	0.0220 (16)	−0.0003 (14)	0.0053 (14)	0.0015 (13)
C3	0.0165 (17)	0.0248 (18)	0.0213 (16)	−0.0005 (14)	0.0027 (13)	0.0021 (14)
C4	0.0201 (18)	0.0217 (18)	0.0241 (16)	−0.0019 (14)	−0.0018 (14)	0.0029 (14)
C5	0.0183 (18)	0.0266 (19)	0.0246 (16)	−0.0005 (15)	0.0056 (14)	0.0027 (15)
C6	0.0216 (18)	0.0265 (19)	0.0213 (16)	0.0017 (15)	0.0046 (14)	0.0018 (14)
C7	0.0191 (17)	0.0177 (17)	0.0164 (14)	0.0001 (14)	0.0036 (12)	0.0019 (13)
C8	0.019 (2)	0.027 (2)	0.0336 (19)	−0.0044 (15)	0.0083 (16)	−0.0056 (16)
C9	0.023 (2)	0.037 (2)	0.037 (2)	−0.0075 (17)	0.0029 (16)	−0.0066 (17)
C10	0.0207 (18)	0.0208 (18)	0.0289 (17)	0.0035 (15)	0.0023 (14)	−0.0007 (14)
C11	0.0152 (17)	0.0191 (18)	0.0204 (15)	0.0002 (13)	0.0034 (13)	0.0018 (13)
C12	0.0189 (17)	0.0221 (18)	0.0219 (15)	−0.0020 (14)	0.0039 (13)	−0.0032 (14)
C13	0.0237 (18)	0.0242 (19)	0.0205 (15)	−0.0010 (15)	0.0055 (13)	−0.0018 (14)
C14	0.0176 (17)	0.0175 (17)	0.0220 (15)	−0.0019 (14)	0.0014 (13)	−0.0008 (13)
C15	0.0187 (17)	0.0177 (17)	0.0215 (15)	0.0024 (13)	0.0037 (13)	0.0000 (13)
C16	0.0180 (16)	0.0172 (16)	0.0174 (14)	0.0004 (14)	0.0046 (12)	−0.0005 (13)
C17	0.0185 (18)	0.0238 (19)	0.0212 (16)	0.0029 (14)	0.0051 (14)	−0.0011 (14)
C18	0.0229 (19)	0.0215 (19)	0.0273 (17)	0.0015 (15)	0.0020 (14)	−0.0015 (14)
C19	0.0200 (17)	0.0168 (17)	0.0218 (15)	−0.0002 (14)	0.0015 (13)	0.0032 (13)
C20	0.0206 (18)	0.0278 (19)	0.0266 (16)	0.0016 (15)	0.0001 (14)	−0.0058 (15)
C21	0.0216 (18)	0.029 (2)	0.0216 (15)	0.0011 (15)	0.0032 (14)	−0.0016 (15)
C22	0.0242 (18)	0.0155 (17)	0.0258 (16)	−0.0011 (14)	−0.0015 (14)	−0.0001 (14)
C23	0.0273 (19)	0.026 (2)	0.0270 (17)	−0.0027 (16)	0.0011 (15)	−0.0014 (15)
C24	0.0243 (18)	0.0207 (18)	0.0311 (17)	−0.0006 (15)	−0.0014 (14)	−0.0005 (15)
C25	0.026 (2)	0.025 (2)	0.0325 (18)	0.0037 (16)	−0.0030 (15)	−0.0027 (16)
C26	0.026 (2)	0.028 (2)	0.0332 (19)	0.0033 (15)	−0.0019 (15)	−0.0047 (16)
C27	0.029 (2)	0.024 (2)	0.0250 (17)	−0.0034 (16)	0.0001 (15)	−0.0007 (15)
C28	0.027 (2)	0.053 (3)	0.039 (2)	−0.0072 (19)	0.0044 (17)	−0.016 (2)
C29	0.031 (2)	0.055 (3)	0.038 (2)	0.002 (2)	−0.0019 (18)	−0.017 (2)
C30	0.0213 (18)	0.0215 (18)	0.0221 (16)	0.0015 (14)	0.0043 (14)	−0.0013 (14)
C31	0.0185 (17)	0.0217 (18)	0.0287 (17)	−0.0021 (14)	0.0045 (14)	−0.0049 (14)
C32	0.0238 (19)	0.0221 (19)	0.0282 (17)	−0.0023 (15)	0.0123 (15)	−0.0010 (15)
C33	0.0251 (19)	0.0262 (19)	0.0221 (16)	−0.0004 (15)	0.0074 (14)	−0.0025 (14)
C34	0.0234 (18)	0.0173 (17)	0.0186 (15)	−0.0005 (14)	0.0063 (13)	−0.0014 (13)
C35	0.0208 (17)	0.0148 (17)	0.0229 (15)	0.0002 (14)	0.0045 (13)	−0.0025 (13)
C36	0.0207 (18)	0.030 (2)	0.0315 (18)	0.0058 (17)	0.0092 (15)	−0.0065 (17)
C37	0.0237 (19)	0.032 (2)	0.040 (2)	−0.0006 (16)	0.0034 (16)	−0.0063 (17)
C38	0.0252 (19)	0.0195 (18)	0.0228 (16)	−0.0020 (14)	0.0039 (14)	−0.0037 (14)
C39	0.0249 (18)	0.0183 (17)	0.0169 (14)	−0.0016 (14)	0.0086 (13)	0.0004 (13)
C40	0.027 (2)	0.034 (2)	0.0231 (17)	−0.0049 (16)	0.0051 (15)	0.0022 (15)
C41	0.029 (2)	0.030 (2)	0.0216 (16)	−0.0045 (16)	0.0048 (15)	0.0032 (15)
C42	0.0266 (19)	0.0165 (17)	0.0236 (16)	0.0016 (14)	0.0066 (14)	0.0017 (14)
C43	0.0229 (17)	0.0190 (18)	0.0190 (15)	0.0013 (14)	0.0059 (13)	0.0004 (13)
C44	0.0236 (18)	0.0178 (17)	0.0209 (15)	0.0010 (14)	0.0057 (14)	−0.0023 (13)
C45	0.0244 (18)	0.0271 (19)	0.0175 (15)	−0.0037 (16)	0.0033 (13)	0.0032 (14)
C46	0.0245 (18)	0.0158 (17)	0.0205 (15)	−0.0009 (14)	0.0056 (13)	0.0023 (13)
C47	0.041 (2)	0.0193 (19)	0.0251 (17)	0.0022 (16)	0.0003 (16)	0.0011 (15)
C48	0.0304 (19)	0.0177 (18)	0.0238 (16)	0.0013 (15)	0.0026 (14)	−0.0007 (14)
C49	0.0236 (18)	0.0265 (19)	0.0294 (17)	0.0019 (16)	0.0020 (14)	−0.0013 (16)
C50	0.0257 (19)	0.0244 (19)	0.0263 (16)	0.0003 (16)	0.0042 (14)	0.0011 (15)
C51	0.032 (2)	0.0235 (19)	0.0245 (17)	0.0075 (16)	−0.0040 (15)	−0.0033 (15)
C52	0.040 (2)	0.035 (2)	0.0294 (18)	0.0081 (18)	−0.0026 (17)	0.0001 (17)
C53	0.035 (2)	0.026 (2)	0.0374 (19)	0.0044 (17)	−0.0083 (16)	−0.0069 (17)
C54	0.035 (2)	0.045 (3)	0.040 (2)	0.0048 (19)	−0.0099 (17)	−0.0055 (19)
C55	0.040 (2)	0.031 (2)	0.0341 (19)	0.0023 (18)	0.0026 (17)	0.0048 (17)
C56	0.032 (2)	0.053 (3)	0.037 (2)	0.000 (2)	0.0018 (17)	0.020 (2)
C57	0.035 (2)	0.050 (3)	0.057 (3)	−0.006 (2)	−0.004 (2)	0.010 (2)
C58	0.038 (3)	0.049 (3)	0.061 (3)	0.006 (2)	0.011 (2)	0.025 (2)

### Geometric parameters (Å, °)

**Table:**

O1—N1	1.422 (3)	C28—H28A	0.9800
O1—H1	0.98 (4)	C28—H28B	0.9800
O2—C8	1.341 (4)	C28—H28C	0.9800
O2—C4	1.464 (3)	C29—H29A	0.9800
O3—C8	1.202 (4)	C29—H29B	0.9800
O4—N2	1.417 (3)	C29—H29C	0.9800
O4—H4	0.95 (4)	C30—C31	1.514 (4)
O5—C36	1.352 (4)	C30—C35	1.533 (4)
O5—C31	1.449 (4)	C30—H30A	0.9900
O6—C36	1.208 (4)	C30—H30B	0.9900
N1—C1	1.270 (4)	C31—C32	1.510 (4)
N2—C46	1.272 (4)	C31—H31	1.0000
C1—C17	1.499 (4)	C32—C33	1.529 (4)
C1—C2	1.504 (4)	C32—H32A	0.9900
C2—C3	1.535 (4)	C32—H32B	0.9900
C2—C7	1.566 (4)	C33—C34	1.536 (4)
C2—H2	1.0000	C33—H33A	0.9900
C3—C4	1.511 (4)	C33—H33B	0.9900
C3—H3A	0.9900	C34—C38	1.536 (4)
C3—H3B	0.9900	C34—C39	1.560 (4)
C4—C5	1.513 (4)	C34—C35	1.561 (4)
C4—H4A	1.0000	C35—C46	1.510 (4)
C5—C6	1.540 (4)	C35—H35	1.0000
C5—H5A	0.9900	C36—C37	1.501 (4)
C5—H5B	0.9900	C37—H37A	0.9800
C6—C7	1.540 (4)	C37—H37B	0.9800
C6—H6A	0.9900	C37—H37C	0.9800
C6—H6B	0.9900	C38—H38A	0.9800
C7—C10	1.539 (4)	C38—H38B	0.9800
C7—C11	1.556 (4)	C38—H38C	0.9800
C8—C9	1.498 (5)	C39—C40	1.521 (4)
C9—H9A	0.9800	C39—C44	1.530 (4)
C9—H9B	0.9800	C39—H39	1.0000
C9—H9C	0.9800	C40—C41	1.539 (4)
C10—H10A	0.9800	C40—H40A	0.9900
C10—H10B	0.9800	C40—H40B	0.9900
C10—H10C	0.9800	C41—C42	1.530 (4)
C11—C12	1.538 (4)	C41—H41A	0.9900
C11—C16	1.542 (4)	C41—H41B	0.9900
C11—H11	1.0000	C42—C47	1.537 (5)
C12—C13	1.545 (4)	C42—C43	1.549 (4)
C12—H12A	0.9900	C42—C48	1.571 (4)
C12—H12B	0.9900	C43—C44	1.524 (4)
C13—C14	1.527 (4)	C43—C50	1.533 (4)
C13—H13A	0.9900	C43—H43	1.0000
C13—H13B	0.9900	C44—C45	1.541 (4)
C14—C15	1.538 (4)	C44—H44	1.0000
C14—C18	1.543 (4)	C45—C46	1.504 (4)
C14—C19	1.562 (4)	C45—H45A	0.9900
C15—C16	1.527 (4)	C45—H45B	0.9900
C15—C21	1.529 (4)	C47—H47A	0.9800
C15—H15	1.0000	C47—H47B	0.9800
C16—C17	1.532 (4)	C47—H47C	0.9800
C16—H16	1.0000	C48—C51	1.539 (4)
C17—H17A	0.9900	C48—C49	1.555 (4)
C17—H17B	0.9900	C48—H48	1.0000
C18—H18A	0.9800	C49—C50	1.542 (4)
C18—H18B	0.9800	C49—H49A	0.9900
C18—H18C	0.9800	C49—H49B	0.9900
C19—C22	1.545 (4)	C50—H50A	0.9900
C19—C20	1.555 (4)	C50—H50B	0.9900
C19—H19	1.0000	C51—C52	1.526 (5)
C20—C21	1.552 (4)	C51—C53	1.537 (5)
C20—H20A	0.9900	C51—H51	1.0000
C20—H20B	0.9900	C52—H52A	0.9800
C21—H21A	0.9900	C52—H52B	0.9800
C21—H21B	0.9900	C52—H52C	0.9800
C22—C24	1.535 (4)	C53—C54	1.530 (4)
C22—C23	1.537 (4)	C53—H53A	0.9900
C22—H22	1.0000	C53—H53B	0.9900
C23—H23A	0.9800	C54—C55	1.537 (5)
C23—H23B	0.9800	C54—H54A	0.9900
C23—H23C	0.9800	C54—H54B	0.9900
C24—C25	1.527 (4)	C55—C56	1.521 (5)
C24—H24A	0.9900	C55—H55A	0.9900
C24—H24B	0.9900	C55—H55B	0.9900
C25—C26	1.529 (4)	C56—C58	1.515 (6)
C25—H25A	0.9900	C56—C57	1.516 (5)
C25—H25B	0.9900	C56—H56	1.0000
C26—C27	1.519 (4)	C57—H57A	0.9800
C26—H26A	0.9900	C57—H57B	0.9800
C26—H26B	0.9900	C57—H57C	0.9800
C27—C29	1.519 (5)	C58—H58A	0.9800
C27—C28	1.525 (5)	C58—H58B	0.9800
C27—H27	1.0000	C58—H58C	0.9800
			
N1—O1—H1	97 (2)	C27—C29—H29C	109.5
C8—O2—C4	117.1 (3)	H29A—C29—H29C	109.5
N2—O4—H4	99 (2)	H29B—C29—H29C	109.5
C36—O5—C31	116.8 (3)	C31—C30—C35	110.3 (2)
C1—N1—O1	113.3 (2)	C31—C30—H30A	109.6
C46—N2—O4	112.8 (2)	C35—C30—H30A	109.6
N1—C1—C17	127.1 (3)	C31—C30—H30B	109.6
N1—C1—C2	118.0 (3)	C35—C30—H30B	109.6
C17—C1—C2	114.9 (3)	H30A—C30—H30B	108.1
C1—C2—C3	114.6 (2)	O5—C31—C32	106.6 (3)
C1—C2—C7	109.6 (2)	O5—C31—C30	110.5 (2)
C3—C2—C7	113.2 (3)	C32—C31—C30	111.8 (3)
C1—C2—H2	106.3	O5—C31—H31	109.3
C3—C2—H2	106.3	C32—C31—H31	109.3
C7—C2—H2	106.3	C30—C31—H31	109.3
C4—C3—C2	109.7 (2)	C31—C32—C33	110.9 (3)
C4—C3—H3A	109.7	C31—C32—H32A	109.5
C2—C3—H3A	109.7	C33—C32—H32A	109.5
C4—C3—H3B	109.7	C31—C32—H32B	109.5
C2—C3—H3B	109.7	C33—C32—H32B	109.5
H3A—C3—H3B	108.2	H32A—C32—H32B	108.0
O2—C4—C3	105.4 (2)	C32—C33—C34	113.3 (3)
O2—C4—C5	109.4 (3)	C32—C33—H33A	108.9
C3—C4—C5	112.0 (3)	C34—C33—H33A	108.9
O2—C4—H4A	110.0	C32—C33—H33B	108.9
C3—C4—H4A	110.0	C34—C33—H33B	108.9
C5—C4—H4A	110.0	H33A—C33—H33B	107.7
C4—C5—C6	111.7 (3)	C33—C34—C38	110.1 (3)
C4—C5—H5A	109.3	C33—C34—C39	110.8 (2)
C6—C5—H5A	109.3	C38—C34—C39	110.5 (3)
C4—C5—H5B	109.3	C33—C34—C35	108.3 (3)
C6—C5—H5B	109.3	C38—C34—C35	110.2 (2)
H5A—C5—H5B	108.0	C39—C34—C35	106.9 (2)
C5—C6—C7	113.3 (2)	C46—C35—C30	114.6 (2)
C5—C6—H6A	108.9	C46—C35—C34	109.8 (2)
C7—C6—H6A	108.9	C30—C35—C34	112.8 (3)
C5—C6—H6B	108.9	C46—C35—H35	106.3
C7—C6—H6B	108.9	C30—C35—H35	106.3
H6A—C6—H6B	107.7	C34—C35—H35	106.3
C10—C7—C6	110.6 (3)	O6—C36—O5	122.6 (3)
C10—C7—C11	111.1 (3)	O6—C36—C37	126.0 (3)
C6—C7—C11	110.3 (2)	O5—C36—C37	111.4 (3)
C10—C7—C2	110.4 (2)	C36—C37—H37A	109.5
C6—C7—C2	106.5 (2)	C36—C37—H37B	109.5
C11—C7—C2	107.8 (2)	H37A—C37—H37B	109.5
O3—C8—O2	123.7 (3)	C36—C37—H37C	109.5
O3—C8—C9	125.4 (3)	H37A—C37—H37C	109.5
O2—C8—C9	110.8 (3)	H37B—C37—H37C	109.5
C8—C9—H9A	109.5	C34—C38—H38A	109.5
C8—C9—H9B	109.5	C34—C38—H38B	109.5
H9A—C9—H9B	109.5	H38A—C38—H38B	109.5
C8—C9—H9C	109.5	C34—C38—H38C	109.5
H9A—C9—H9C	109.5	H38A—C38—H38C	109.5
H9B—C9—H9C	109.5	H38B—C38—H38C	109.5
C7—C10—H10A	109.5	C40—C39—C44	111.4 (3)
C7—C10—H10B	109.5	C40—C39—C34	114.5 (3)
H10A—C10—H10B	109.5	C44—C39—C34	112.2 (2)
C7—C10—H10C	109.5	C40—C39—H39	106.0
H10A—C10—H10C	109.5	C44—C39—H39	106.0
H10B—C10—H10C	109.5	C34—C39—H39	106.0
C12—C11—C16	110.2 (2)	C39—C40—C41	113.9 (3)
C12—C11—C7	113.4 (2)	C39—C40—H40A	108.8
C16—C11—C7	113.3 (2)	C41—C40—H40A	108.8
C12—C11—H11	106.5	C39—C40—H40B	108.8
C16—C11—H11	106.5	C41—C40—H40B	108.8
C7—C11—H11	106.5	H40A—C40—H40B	107.7
C11—C12—C13	113.5 (3)	C42—C41—C40	111.3 (3)
C11—C12—H12A	108.9	C42—C41—H41A	109.4
C13—C12—H12A	108.9	C40—C41—H41A	109.4
C11—C12—H12B	108.9	C42—C41—H41B	109.4
C13—C12—H12B	108.9	C40—C41—H41B	109.4
H12A—C12—H12B	107.7	H41A—C41—H41B	108.0
C14—C13—C12	111.6 (2)	C41—C42—C47	111.3 (3)
C14—C13—H13A	109.3	C41—C42—C43	107.0 (2)
C12—C13—H13A	109.3	C47—C42—C43	111.8 (3)
C14—C13—H13B	109.3	C41—C42—C48	116.8 (3)
C12—C13—H13B	109.3	C47—C42—C48	109.9 (3)
H13A—C13—H13B	108.0	C43—C42—C48	99.4 (2)
C13—C14—C15	107.3 (2)	C44—C43—C50	119.1 (2)
C13—C14—C18	110.6 (3)	C44—C43—C42	114.3 (3)
C15—C14—C18	112.0 (3)	C50—C43—C42	103.7 (2)
C13—C14—C19	116.7 (2)	C44—C43—H43	106.3
C15—C14—C19	100.3 (2)	C50—C43—H43	106.3
C18—C14—C19	109.6 (3)	C42—C43—H43	106.3
C16—C15—C21	118.7 (2)	C43—C44—C39	110.2 (2)
C16—C15—C14	113.5 (2)	C43—C44—C45	110.2 (3)
C21—C15—C14	104.3 (2)	C39—C44—C45	110.4 (3)
C16—C15—H15	106.5	C43—C44—H44	108.6
C21—C15—H15	106.5	C39—C44—H44	108.6
C14—C15—H15	106.5	C45—C44—H44	108.6
C15—C16—C17	111.2 (2)	C46—C45—C44	112.6 (3)
C15—C16—C11	109.1 (2)	C46—C45—H45A	109.1
C17—C16—C11	111.9 (2)	C44—C45—H45A	109.1
C15—C16—H16	108.2	C46—C45—H45B	109.1
C17—C16—H16	108.2	C44—C45—H45B	109.1
C11—C16—H16	108.2	H45A—C45—H45B	107.8
C1—C17—C16	109.1 (3)	N2—C46—C45	125.6 (3)
C1—C17—H17A	109.9	N2—C46—C35	117.9 (3)
C16—C17—H17A	109.9	C45—C46—C35	116.5 (2)
C1—C17—H17B	109.9	C42—C47—H47A	109.5
C16—C17—H17B	109.9	C42—C47—H47B	109.5
H17A—C17—H17B	108.3	H47A—C47—H47B	109.5
C14—C18—H18A	109.5	C42—C47—H47C	109.5
C14—C18—H18B	109.5	H47A—C47—H47C	109.5
H18A—C18—H18B	109.5	H47B—C47—H47C	109.5
C14—C18—H18C	109.5	C51—C48—C49	112.1 (3)
H18A—C18—H18C	109.5	C51—C48—C42	119.6 (3)
H18B—C18—H18C	109.5	C49—C48—C42	102.8 (2)
C22—C19—C20	112.1 (3)	C51—C48—H48	107.2
C22—C19—C14	118.3 (3)	C49—C48—H48	107.2
C20—C19—C14	103.1 (2)	C42—C48—H48	107.2
C22—C19—H19	107.6	C50—C49—C48	107.7 (3)
C20—C19—H19	107.6	C50—C49—H49A	110.2
C14—C19—H19	107.6	C48—C49—H49A	110.2
C21—C20—C19	107.2 (2)	C50—C49—H49B	110.2
C21—C20—H20A	110.3	C48—C49—H49B	110.2
C19—C20—H20A	110.3	H49A—C49—H49B	108.5
C21—C20—H20B	110.3	C43—C50—C49	103.3 (2)
C19—C20—H20B	110.3	C43—C50—H50A	111.1
H20A—C20—H20B	108.5	C49—C50—H50A	111.1
C15—C21—C20	103.6 (2)	C43—C50—H50B	111.1
C15—C21—H21A	111.0	C49—C50—H50B	111.1
C20—C21—H21A	111.0	H50A—C50—H50B	109.1
C15—C21—H21B	111.0	C52—C51—C53	109.9 (3)
C20—C21—H21B	111.0	C52—C51—C48	113.2 (3)
H21A—C21—H21B	109.0	C53—C51—C48	108.4 (3)
C24—C22—C23	109.9 (3)	C52—C51—H51	108.4
C24—C22—C19	110.8 (3)	C53—C51—H51	108.4
C23—C22—C19	112.8 (3)	C48—C51—H51	108.4
C24—C22—H22	107.7	C51—C52—H52A	109.5
C23—C22—H22	107.7	C51—C52—H52B	109.5
C19—C22—H22	107.7	H52A—C52—H52B	109.5
C22—C23—H23A	109.5	C51—C52—H52C	109.5
C22—C23—H23B	109.5	H52A—C52—H52C	109.5
H23A—C23—H23B	109.5	H52B—C52—H52C	109.5
C22—C23—H23C	109.5	C54—C53—C51	116.5 (3)
H23A—C23—H23C	109.5	C54—C53—H53A	108.2
H23B—C23—H23C	109.5	C51—C53—H53A	108.2
C25—C24—C22	114.3 (3)	C54—C53—H53B	108.2
C25—C24—H24A	108.7	C51—C53—H53B	108.2
C22—C24—H24A	108.7	H53A—C53—H53B	107.3
C25—C24—H24B	108.7	C53—C54—C55	113.1 (3)
C22—C24—H24B	108.7	C53—C54—H54A	109.0
H24A—C24—H24B	107.6	C55—C54—H54A	109.0
C24—C25—C26	112.8 (3)	C53—C54—H54B	109.0
C24—C25—H25A	109.0	C55—C54—H54B	109.0
C26—C25—H25A	109.0	H54A—C54—H54B	107.8
C24—C25—H25B	109.0	C56—C55—C54	114.0 (3)
C26—C25—H25B	109.0	C56—C55—H55A	108.8
H25A—C25—H25B	107.8	C54—C55—H55A	108.8
C27—C26—C25	115.1 (3)	C56—C55—H55B	108.8
C27—C26—H26A	108.5	C54—C55—H55B	108.8
C25—C26—H26A	108.5	H55A—C55—H55B	107.7
C27—C26—H26B	108.5	C58—C56—C57	110.1 (3)
C25—C26—H26B	108.5	C58—C56—C55	110.3 (3)
H26A—C26—H26B	107.5	C57—C56—C55	111.9 (3)
C29—C27—C26	113.2 (3)	C58—C56—H56	108.1
C29—C27—C28	110.2 (3)	C57—C56—H56	108.1
C26—C27—C28	110.6 (3)	C55—C56—H56	108.1
C29—C27—H27	107.5	C56—C57—H57A	109.5
C26—C27—H27	107.5	C56—C57—H57B	109.5
C28—C27—H27	107.5	H57A—C57—H57B	109.5
C27—C28—H28A	109.5	C56—C57—H57C	109.5
C27—C28—H28B	109.5	H57A—C57—H57C	109.5
H28A—C28—H28B	109.5	H57B—C57—H57C	109.5
C27—C28—H28C	109.5	C56—C58—H58A	109.5
H28A—C28—H28C	109.5	C56—C58—H58B	109.5
H28B—C28—H28C	109.5	H58A—C58—H58B	109.5
C27—C29—H29A	109.5	C56—C58—H58C	109.5
C27—C29—H29B	109.5	H58A—C58—H58C	109.5
H29A—C29—H29B	109.5	H58B—C58—H58C	109.5
			
O1—N1—C1—C17	−1.5 (4)	C36—O5—C31—C32	156.6 (3)
O1—N1—C1—C2	−179.0 (2)	C36—O5—C31—C30	−81.7 (3)
N1—C1—C2—C3	−11.2 (4)	C35—C30—C31—O5	−175.1 (3)
C17—C1—C2—C3	171.0 (3)	C35—C30—C31—C32	−56.5 (4)
N1—C1—C2—C7	117.3 (3)	O5—C31—C32—C33	177.2 (2)
C17—C1—C2—C7	−60.5 (3)	C30—C31—C32—C33	56.3 (3)
C1—C2—C3—C4	−174.8 (3)	C31—C32—C33—C34	−56.0 (4)
C7—C2—C3—C4	58.6 (4)	C32—C33—C34—C38	−66.9 (3)
C8—O2—C4—C3	−158.8 (3)	C32—C33—C34—C39	170.5 (3)
C8—O2—C4—C5	80.7 (3)	C32—C33—C34—C35	53.6 (3)
C2—C3—C4—O2	−174.7 (2)	C31—C30—C35—C46	−177.3 (3)
C2—C3—C4—C5	−55.9 (3)	C31—C30—C35—C34	56.1 (4)
O2—C4—C5—C6	171.0 (3)	C33—C34—C35—C46	177.0 (3)
C3—C4—C5—C6	54.6 (4)	C38—C34—C35—C46	−62.5 (3)
C4—C5—C6—C7	−55.3 (4)	C39—C34—C35—C46	57.6 (3)
C5—C6—C7—C10	−65.6 (3)	C33—C34—C35—C30	−53.9 (3)
C5—C6—C7—C11	171.1 (3)	C38—C34—C35—C30	66.6 (3)
C5—C6—C7—C2	54.3 (3)	C39—C34—C35—C30	−173.2 (2)
C1—C2—C7—C10	−65.9 (3)	C31—O5—C36—O6	−2.6 (4)
C3—C2—C7—C10	63.4 (3)	C31—O5—C36—C37	178.0 (3)
C1—C2—C7—C6	174.0 (2)	C33—C34—C39—C40	52.2 (4)
C3—C2—C7—C6	−56.7 (3)	C38—C34—C39—C40	−70.1 (3)
C1—C2—C7—C11	55.6 (3)	C35—C34—C39—C40	169.9 (3)
C3—C2—C7—C11	−175.1 (2)	C33—C34—C39—C44	−179.7 (3)
C4—O2—C8—O3	3.8 (5)	C38—C34—C39—C44	58.0 (3)
C4—O2—C8—C9	−175.1 (3)	C35—C34—C39—C44	−61.9 (3)
C10—C7—C11—C12	−59.8 (3)	C44—C39—C40—C41	52.1 (4)
C6—C7—C11—C12	63.1 (3)	C34—C39—C40—C41	−179.3 (3)
C2—C7—C11—C12	179.1 (3)	C39—C40—C41—C42	−55.7 (4)
C10—C7—C11—C16	66.7 (3)	C40—C41—C42—C47	−66.6 (3)
C6—C7—C11—C16	−170.3 (3)	C40—C41—C42—C43	55.8 (4)
C2—C7—C11—C16	−54.4 (3)	C40—C41—C42—C48	166.0 (3)
C16—C11—C12—C13	52.9 (3)	C41—C42—C43—C44	−58.7 (3)
C7—C11—C12—C13	−178.9 (3)	C47—C42—C43—C44	63.4 (4)
C11—C12—C13—C14	−54.6 (4)	C48—C42—C43—C44	179.4 (3)
C12—C13—C14—C15	55.3 (3)	C41—C42—C43—C50	170.1 (3)
C12—C13—C14—C18	−67.1 (3)	C47—C42—C43—C50	−67.9 (3)
C12—C13—C14—C19	166.8 (3)	C48—C42—C43—C50	48.2 (3)
C13—C14—C15—C16	−60.4 (3)	C50—C43—C44—C39	−179.9 (3)
C18—C14—C15—C16	61.1 (3)	C42—C43—C44—C39	56.8 (3)
C19—C14—C15—C16	177.2 (2)	C50—C43—C44—C45	−57.7 (4)
C13—C14—C15—C21	169.0 (2)	C42—C43—C44—C45	179.0 (3)
C18—C14—C15—C21	−69.5 (3)	C40—C39—C44—C43	−51.1 (4)
C19—C14—C15—C21	46.6 (3)	C34—C39—C44—C43	179.1 (3)
C21—C15—C16—C17	−52.5 (4)	C40—C39—C44—C45	−173.1 (3)
C14—C15—C16—C17	−175.6 (3)	C34—C39—C44—C45	57.1 (3)
C21—C15—C16—C11	−176.4 (3)	C43—C44—C45—C46	−169.9 (3)
C14—C15—C16—C11	60.5 (3)	C39—C44—C45—C46	−47.9 (4)
C12—C11—C16—C15	−54.2 (3)	O4—N2—C46—C45	1.4 (5)
C7—C11—C16—C15	177.6 (3)	O4—N2—C46—C35	−179.9 (3)
C12—C11—C16—C17	−177.7 (3)	C44—C45—C46—N2	−133.0 (3)
C7—C11—C16—C17	54.1 (3)	C44—C45—C46—C35	48.3 (4)
N1—C1—C17—C16	−119.9 (3)	C30—C35—C46—N2	−0.7 (4)
C2—C1—C17—C16	57.7 (4)	C34—C35—C46—N2	127.5 (3)
C15—C16—C17—C1	−174.7 (3)	C30—C35—C46—C45	178.2 (3)
C11—C16—C17—C1	−52.4 (3)	C34—C35—C46—C45	−53.7 (4)
C13—C14—C19—C22	80.5 (4)	C41—C42—C48—C51	80.1 (4)
C15—C14—C19—C22	−164.0 (3)	C47—C42—C48—C51	−47.8 (4)
C18—C14—C19—C22	−46.1 (4)	C43—C42—C48—C51	−165.3 (3)
C13—C14—C19—C20	−155.1 (3)	C41—C42—C48—C49	−155.0 (3)
C15—C14—C19—C20	−39.7 (3)	C47—C42—C48—C49	77.1 (3)
C18—C14—C19—C20	78.3 (3)	C43—C42—C48—C49	−40.4 (3)
C22—C19—C20—C21	147.6 (3)	C51—C48—C49—C50	148.8 (3)
C14—C19—C20—C21	19.3 (3)	C42—C48—C49—C50	19.1 (3)
C16—C15—C21—C20	−162.2 (3)	C44—C43—C50—C49	−165.0 (3)
C14—C15—C21—C20	−34.7 (3)	C42—C43—C50—C49	−36.6 (3)
C19—C20—C21—C15	9.0 (4)	C48—C49—C50—C43	10.3 (4)
C20—C19—C22—C24	55.8 (3)	C49—C48—C51—C52	−176.2 (3)
C14—C19—C22—C24	175.6 (3)	C42—C48—C51—C52	−55.8 (4)
C20—C19—C22—C23	179.5 (3)	C49—C48—C51—C53	61.6 (4)
C14—C19—C22—C23	−60.6 (4)	C42—C48—C51—C53	−178.1 (3)
C23—C22—C24—C25	73.6 (4)	C52—C51—C53—C54	64.8 (4)
C19—C22—C24—C25	−161.0 (3)	C48—C51—C53—C54	−171.0 (3)
C22—C24—C25—C26	168.9 (3)	C51—C53—C54—C55	65.8 (4)
C24—C25—C26—C27	−167.5 (3)	C53—C54—C55—C56	165.0 (3)
C25—C26—C27—C29	−65.6 (4)	C54—C55—C56—C58	168.9 (3)
C25—C26—C27—C28	170.2 (3)	C54—C55—C56—C57	−68.2 (4)

### Hydrogen-bond geometry (Å, °)

**Table:**

*D*—H···*A*	*D*—H	H···*A*	*D*···*A*	*D*—H···*A*
O1—H1···N2	0.98 (4)	1.88 (4)	2.809 (3)	157 (4)
O4—H4···N1	0.95 (4)	1.82 (4)	2.733 (3)	160 (3)
C9—H9c···O6^i^	0.98	2.58	3.404 (4)	142
C37—H37c···O3^ii^	0.98	2.40	3.373 (4)	169

Symmetry codes: (i) *x*+1, *y*, *z*; (ii) −*x*+2, *y*−1/2, −*z*+1.
